# Supplementary material for: Mechanisms governing the pioneering and redistribution capabilities of the non-classical pioneer PU.1
Source: Nat Commun. 2020 Jan 21;11:402. doi: 10.1038/s41467-019-13960-2 (PMC6972792; doi:10.1038/s41467-019-13960-2)
Supplement: Supplementary file 7 — Source data [file 41467_2019_13960_MOESM7_ESM.zip › Source_Data/FigureS5/FigureS5B_MotifsETS1specificPeaks/homerResults/motif2.similar.html]

motif2

## Information for motif2

T
G
A
C
T
G
C
A
C
T
A
G
C
T
A
G
C
G
T
A
A
C
G
T
A
C
T
G
A
G
C
T
A
C
T
G
T
C
A
G
A
C
G
T
G
A
T
C
C
G
T
A
  
Reverse Opposite:  

G
C
A
T
C
T
A
G
T
G
C
A
A
G
T
C
G
T
A
C
C
T
G
A
A
G
T
C
C
G
T
A
C
G
A
T
A
G
T
C
A
G
T
C
A
C
G
T
A
C
T
G
  

|  |  |
| --- | --- |
| p-value: | 1e-179 |
| log p-value: | -4.129e+02 |
| Information Content per bp: | 1.698 |
| Number of Target Sequences with motif | 364.0 |
| Percentage of Target Sequences with motif | 32.07% |
| Number of Background Sequences with motif | 279.3 |
| Percentage of Background Sequences with motif | 3.42% |
| Average Position of motif in Targets | 102.2 +/- 24.3bp |
| Average Position of motif in Background | 99.5 +/- 43.9bp |
| Strand Bias (log2 ratio + to - strand density) | 0.3 |
| Multiplicity (# of sites on avg that occur together) | 1.01 |
| Motif File: | file (matrix) reverse opposite |

### Similar de novo motifs found

|  |  |  |  |  |  |  |  |
| --- | --- | --- | --- | --- | --- | --- | --- |
| Rank | Match Score | Redundant Motif | P-value | log P-value | % of Targets | % of Background | Motif file |
| 1 | 0.961 | T G C A C A T G A T C G C T G A A C G T C T A G G A C T A C T G A T C G C A G T A G T C G C A T | 1e-177 | -408.077357 | 32.78% | 3.76% | motif file (matrix) |
| 2 | 0.964 | C T A G T A G C C G T A A C T G A C T G C G T A A C G T A C T G A G C T A C T G A C T G A C G T A G C T C G A T | 1e-167 | -385.591527 | 29.25% | 2.92% | motif file (matrix) |
| 3 | 0.953 | T C G A C A T G C A T G C T G A A C G T A C T G G A C T A C T G A T C G C A G T G A C T | 1e-166 | -382.510330 | 31.89% | 3.89% | motif file (matrix) |
| 4 | 0.961 | A G T C A T G C C G T A A G T C C G T A A C G T A G T C G T A C G A C T A T C G | 1e-163 | -376.989328 | 29.78% | 3.23% | motif file (matrix) |
| 5 | 0.954 | A G T C A G T C C T G A A G T C C G T A A C G T A G T C A T G C A C G T | 1e-162 | -373.533595 | 30.31% | 3.47% | motif file (matrix) |
| 6 | 0.945 | A C T G A C T G C G T A A C G T A C T G A C G T A C T G A C T G | 1e-152 | -351.176438 | 34.27% | 5.50% | motif file (matrix) |
| 7 | 0.855 | C G T A A G T C C G T A A C G T A G T C A G T C A C G T | 1e-125 | -288.071452 | 35.77% | 7.90% | motif file (matrix) |
| 8 | 0.753 | A C T G A T C G G T C A A G T C C T A G G A C T A C T G C T A G A C G T | 1e-105 | -243.193982 | 20.79% | 2.45% | motif file (matrix) |
| 9 | 0.858 | T G C A C T G A A G T C A G T C C T A G A G T C G T C A A C G T A T G C A G T C | 1e-91 | -209.769292 | 18.59% | 2.31% | motif file (matrix) |
| 10 | 0.835 | G T A C T G C A G T C A C T G A A G T C C G T A A C G T A G T C A G T C C A G T A T C G | 1e-79 | -182.899602 | 16.74% | 2.18% | motif file (matrix) |
| 11 | 0.810 | G C T A T A G C G C T A C T G A A T G C G C T A C G A T A G T C A T G C A G C T A C T G A G T C | 1e-79 | -181.916648 | 18.50% | 2.87% | motif file (matrix) |
| 12 | 0.813 | C G T A T G C A A G T C G T A C C T G A A G T C C G T A C G A T A G T C A G T C C G A T C A T G A T C G | 1e-30 | -70.319283 | 5.64% | 0.56% | motif file (matrix) |
| 13 | 0.799 | G C A T G T A C A G C T A G T C A G T C G C T A A G T C G T C A A C G T A G T C A G T C A C T G T G A C | 1e-14 | -33.753753 | 2.73% | 0.29% | motif file (matrix) |
| 14 | 0.767 | A G T C C T G A A C T G A C T G C G T A A C G T A C T G C G A T A C G T | 1e-14 | -32.744454 | 3.88% | 0.73% | motif file (matrix) |
